# Association Between Trp64Arg Polymorphism of Beta-3 Adrenergic Receptor Gene and Susceptibility to Overactive Bladder: A Meta-Analysis

**DOI:** 10.3389/fgene.2022.930084

**Published:** 2022-07-12

**Authors:** Rong Dai, Yue Chen, Kai Yang, Tao Wu, Changkai Deng

**Affiliations:** ^1^ Chengdu Center for Disease Control and Prevention, Chengdu, China; ^2^ Chenghua Center for Disease Control and Prevention, Chengdu, China; ^3^ Chengdu Women’s and Children’s Central Hospital, School of Medicine, University of Electronic Science and Technology of China, Chengdu, China

**Keywords:** beta-3 adrenergic receptor gene, meta-analysis, overactive bladder, polymorphism, trail sequential analysis running head: relationship between overactive bladder and Trp64Arg polymorphism

## Abstract

**Objective:** Some studies have been carried out to investigate the association between Trp64Arg polymorphism in beta-3 adrenergic receptor gene (*ADRB3*) and susceptibility to overactive bladder (OAB), but the results remain inconsistent. We carried out a meta-analysis to acquire a more accurate estimation.

**Methods:** All eligible studies were searched in PubMed, Web of Science, Embase, and Cochrane Library. Pooled odds ratios, with 95% confidence intervals, were assessed for the association using fixed and random effects models.

**Results:** The overall results of this meta-analysis demonstrated that there might be an association between Trp64Arg polymorphism and susceptibility to OAB in allele model, dominant model, and heterozygote comparison with a relative risk of 2.00 (95% CI 1.36–2.93), 2.13 (95% CI 1.20–3.76), and 2.07 (95% CI: 1.13–3.79), respectively. However, in the recessive model and homozygote comparison, no significant association between *ESR1* Trp64Arg polymorphism and susceptibility to OAB was observed, with a relative risk of 2.47 (95% CI 0.63–9.73) and 3.12 (95% CI: 0.79–12.35), respectively. Based on trail sequential analysis, the results turned out to be true positive in the allele model, false positive in the dominant model and heterozygote comparison, and negative in the recessive model and homozygote comparison, respectively.

**Conclusion:** Our analysis indicated that Trp64Arg polymorphisms in *ADRB3* might increase the risk of OAB twice in the allele model, but further well-designed studies with large sample sizes are required to confirm the present findings in other modes and comparisons.

## Introduction

Overactive bladder (OAB) is a common clinical condition with urinary urgency, accompanied by frequency, nocturia, and urinary incontinence, without other neurological conditions or urinary tract infection (Nevéus et al., 2006). OAB is a harmful syndrome that can have its onset in childhood and persist into adulthood (Salvatore et al., 2012). Published data indicate that these individuals are more likely to have depression, anxiety, and attention deficit problems (Franco, 2016).

OAB is a storage symptom syndrome with multiple possible pathogeneses (Funada et al., 2018; Peyronnet et al., 2019). Recognition of the mechanisms would help tailor diagnosis and therapy to individual patients and improve prognosis (Peyronnet et al., 2019). The detrusor muscle contains beta-3 adrenergic receptor (beta-AR) subtypes (beta1-AR, beta2-AR, and beta3-AR) (Yamaguchi, 2002). Present evidence suggests that beta3-AR, encoded by the beta3-AR gene (*ADRB3*), is a predominant subtype (with 97% of the total) in human bladder tissue and is implicated in metabolic functions of endogenous catecholamines, leading to bladder relaxation (Yamaguchi, 2002; Yamaguchi and Chapple, 2007). Previous studies of families and twins have suggested a genetic predisposition to OAB (Rohr et al., 2004; Wennberg et al., 2011). Trp64Arg polymorphism (ID rs4994) of *ADRB3* leads to a missense mutation resulting in an amino acid change from tryptophan to arginine and altering receptor function, which has been proposed as a pathophysiologic mechanism for OAB (Michel and Korstanje, 2016; Meekins et al., 2019).

Two studies concluded that the Trp64Arg polymorphism was associated with OAB in Brazilian and Japanese populations (Ferreira et al., 2011; Honda et al., 2014). Similarly, two meta-analyses provided moderate credibility for associations of the Trp64Arg polymorphism with OAB (Cartwright et al., 2015; Qu et al., 2015). Meanwhile, another three studies indicated that the Trp64Arg polymorphism was not susceptible to OAB, but polymorphic patients seemed to suffer from clinical disappointment in the Turkish population (Gurocak et al., 2015; Fırat et al., 2020; Çırakoğlu et al., 2021).

There are only a few original studies on the relationship between Trp64Arg polymorphism and OAB; moreover, the results are contradictory and available evidence remains limited (Yamaguchi, 2002; Ferreira et al., 2011; Teitsma et al., 2013; Honda et al., 2014; Gurocak et al., 2015; Meekins et al., 2019; Fırat et al., 2020; Çırakoğlu et al., 2021). In order to overcome the limitations of individual study and acquire a more accurate estimation of the association between Trp64Arg polymorphism and susceptibility to OAB, this meta-analysis was performed.

## Methods

### Data Source

We searched four databases, PubMed, Web of Science, Embase, and Cochrane Library, until 23 March 2022. The search was based on the keywords as follows: “overactive bladder” or “OAB” combined with “genetic polymorphisms” or “SNP” or “beta-3 adrenergic receptors” or “ADRB3” or “rs4994” or “B3-AR” or “Trp64Arg”. Our review was based on the Preferred Reporting Items for Systematic Reviews (PRISMA) guidelines (Moher et al., 2009). The detail is presented in Supplementary Table S1.

### Inclusion Criteria and Exclusion Criteria

Our inclusion criteria were as follows: 1) the study explored the association between *ADRB3* gene polymorphism and susceptibility to OAB; 2) the *ADRB3* Trp64Arg polymorphism was tested in the study; 3) the articles identified the sample size of case and control groups and the distribution of alleles and (or) genotypes; 4) publication language was English. 5) genotypes distribution met Hardy–Weinberg equilibrium (HWE) in the control group (Stark and Seneta, 2013).

Studies were excluded if they were editorials, letters, meeting abstracts, reviews, or if they reported no target or incomplete data or no relevant outcomes or overlapping data.

### Data Extraction

Two reviewers (Rong Dai and Yue Chen) independently searched the literature and extracted data. Disagreements were solved by discussion, and a third party (Kai Yang and Tao Wu) was involved when necessary. The items included corresponding author, date of publication, setting, ethnicity, sample size of case and control, gender, age, specimen source, study design, and genotyping method.

### Quality Assessment

The Newcastle–Ottawa Quality Assessment Scale (NOS) was used for a quality assessment of the included articles. Scores of 7–9, 4–6, and 0–3 were assigned as high-, moderate-, and low-quality studies, respectively.

### Statistical Analyses

The deviation of HWE in the control group was tested by the goodness-of-fit chi-square test. The association between Trp64Arg polymorphism and susceptibility to OAB was analyzed by calculating the odds ratio (OR) with a 95% confidence interval (CI). Heterogeneity among included studies was checked by chi-square-based Q test and I^2^ test. If the data showed no heterogeneity (*p* > 0.05, I^2^ = 0%), the fixed effect model was used; otherwise, the random effect model was used. The pooled ORs were performed for the allele model (C vs. T), dominant model (CC + TC vs. TT), recessive model (CC vs. TT + TC), homozygote comparison (CC vs. TT), and heterozygote comparison (TC vs. TT). All statistical tests were conducted with the Stata 12.0. A *p*-value of 0.05 for any test or model was considered statistically significant unless otherwise specified.

### Publication Bias

Potential publication bias was assessed by the Egger linear regression test using the software of Stata 12.0. The genotypes that were included in more than two studies were tested.

### Trail Sequential Analysis

Trail sequential analysis (TSA) revealed insufficient information size and potentially false positive results in many meta-analyses. Therefore, the threshold for statistical significance was adjusted by trail sequential analysis (an overall 5% risk of a type I error and 20% of the type II error) when original studies were sparse. Another research study we have published should be referred to (Deng et al., 2016).

## Results

### Literature Selection

**FIGURE 1:**
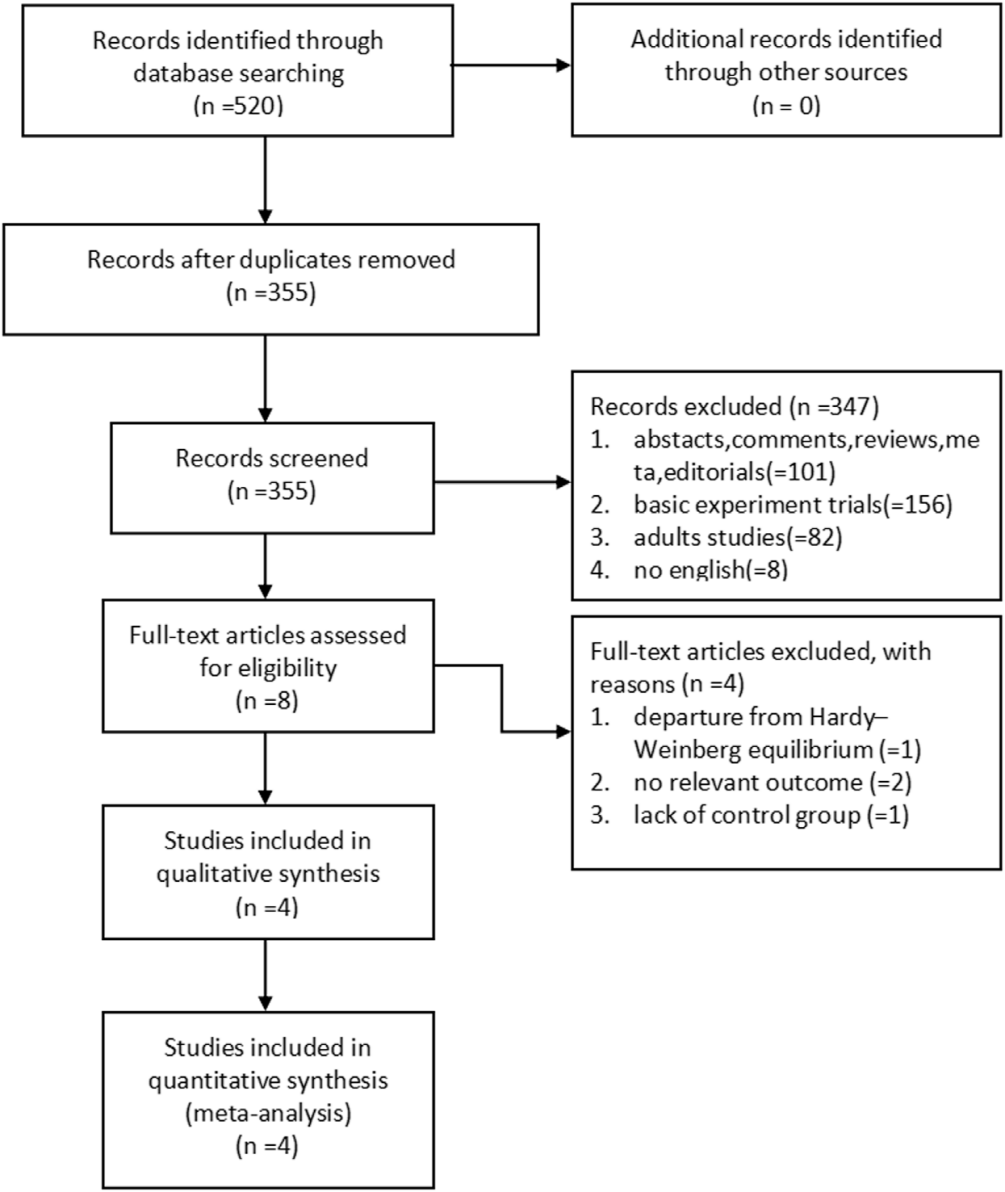
Flow chart of literature selection.

We initially identified 520 potentially eligible studies. Most of them were excluded after the screening of titles and abstracts. The main reason for excluding was duplication and no association between *ADRB3* gene polymorphism and susceptibility to OAB. After assessing the full text of eight potentially relevant articles, we identified four eligible articles with high scores of NOS (Table 1). The main reasons for exclusion were as follows: one study deviated from HWE in the control group, two studies had no relevant outcome, and one study had a lack of control group (Figure 1).

**TABLE 1:** Characteristic of the included studies.

Corresponding author	Year	Setting	Ethnicity	Sample size (T/C)		Gender	Age (year)	Sample	Design	Genotyping method	Score
				Case	Control						
Abdullah	2021	Turkey	Turkish	131/13	144/12	Female/male	30–87	Blood	Case-control	PCR-RFLP	8
Gurocak S	2015	Turkey	Turkish	64/4	79/5	Female/male	>5	Blood	Case-control	Real-Time PCR	7
Honda K	2014	Japan	Japanese	148/52	177/25	Female	>18	Hair	Case-control	Real-Time PCR	7
Fonseca AM	2011	Brazil	Brazilian	73/25	296/42	Female	>18^∗^	Blood	Case-control	Real-Time PCR	8

^∗^Age of the control groups was ≥60. T/C, case group/control group.

### Study Characteristics

Four studies with 255 cases and 390 controls were included in the analysis. The basic characteristics of included studies are presented in Table 1. All studies were published between 2011 and 2021. The included studies were conducted in Japan, Brazil, and Turkey, respectively. Moreover, the studies involved Japanese, Brazilian, and Turkish populations. All the included studies were case-control studies. The distribution of *ADRB3* SNP64 T > C genotype among OAB cases and controls of the four studies are listed in Supplementary Table S2, and the genotype distribution in the controls of all included studies was consistent with HWE (Supplementary Table S2).

### Meta-Analysis Results

**FIGURE 2:**
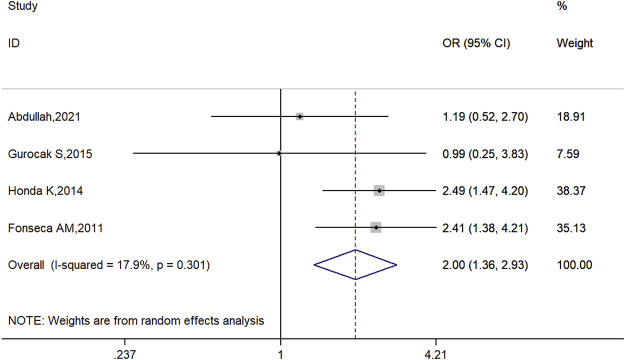
Forest plots for the association between *ADRB3* gene polymorphism and susceptibility to OAB in the allele model (C vs. T).

The overall results of this meta-analysis demonstrated that there may be an association between *ADRB3* Trp64Arg polymorphism and susceptibility to OAB in allele model (C vs. T: OR = 2.00, 95% CI: 1.36–2.93, *p* = 0.00; Figure 2), dominant model (CC + TC vs. TT: OR = 2.13, 95% CI: 1.20–3.76, *p* = 0.01; Supplementary Figure S1), and heterozygote comparison (TC vs. TT: OR = 2.07, 95% CI: 1.13–3.79, *p* = 0.02; Supplementary Figure S2) and may be no significant association between *ADRB3* Trp64Arg polymorphism and susceptibility to OAB in recessive model (CC vs. TT + TC: OR = 2.47, 95% CI: 0.63–9.73, *p* = 0.20; Supplementary Figure S3) and homozygote comparison (CC vs. TT: OR = 3.12, 95% CI: 0.79–12.35, *p* = 0.11; Supplementary Figure S4). The details are listed in Table 2.

**TABLE 2:** Results of the meta-analysis.

Genetic model	MD (95%CI)	*Z*	*P-*Value	*I* ^*2*^ *%*	*P*_*het*_	Egger’s test	
						*t*	*P-*Value
C:T	2.00 (1.36, 2.93)	3.52	0.00	17.9	0.30	−2.65	0.12
CC:TT	3.12 (0.79,12.35)	1.62	0.11	0.00	0.73	-	-
TC:TT	2.07 (1.13, 3.79)	2.36	0.02	51.10	0.11	−2.41	0.14
CC:TC + TT	2.47 (0.63,9.73)	1.29	0.20	0.00	0.91	-	-
CC + TC:TT	2.13 (1.20, 3.76)	2.60	0.01	47.80	0.12	−2.19	0.16

### Publication Bias

The results of Egger’s linear regression test supported the conclusion of no significant publication bias in heterozygote comparison, dominant model, and allele model (Table 2).

### Trail Sequential Analysis

**FIGURE 3:**
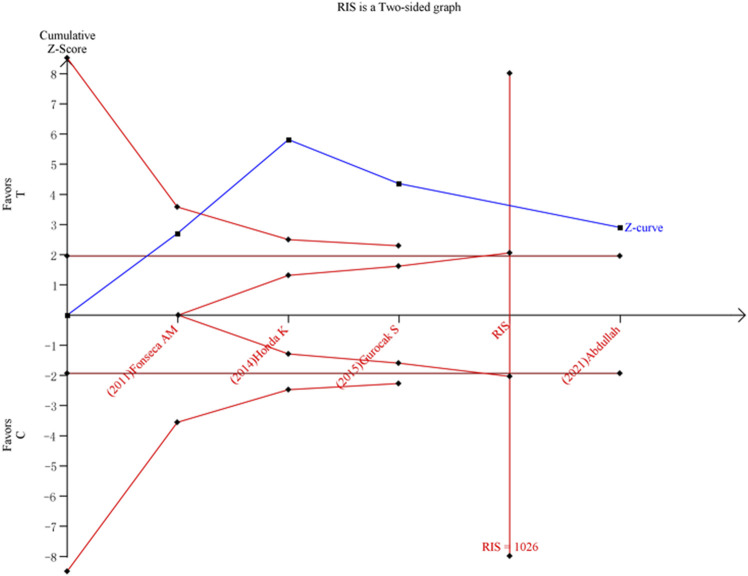
Result of TSA for the association between *ADRB3* gene polymorphism and susceptibility to OAB in the allele model (C vs. T).

We calculated the required information size to 1026, 3135, 4707, 5103334, and 1684188 patients, respectively, for allele model, dominant model, heterozygote comparison, homozygote comparison, and recessive model. Moreover, we found that C allele as a risk factor turned out to be true positive in the allele model (Figure 3), but the results in the dominant model and heterozygote comparison turned out to be false positive (Supplementary Figures S5 and S6), and the results in the recessive model and homozygote comparison turned out to be negative (Supplementary Figures S7 and S8).

## Discussion

Genetic correlation studies are used to evaluate the underlying association between genotype and phenotype occurring in a population (Nedumaran et al., 2019). Previous studies of families and twins have suggested a hereditary susceptibility to OAB (Rohr et al., 2004; Wennberg et al., 2011). Igawa et al. provided the first evidence for the existence of the beta3-AR subtype in the human detrusor and suggested that the relaxation induced by parasympathetic nervous systems of the human detrusor is mediated mainly through beta3-AR activation (Igawa et al., 1999). Although several factors seem to be involved in the mechanisms underlying OAB, present studies may suggest that dysfunction of beta3-AR resulting from its gene polymorphism, at least partially, conduces to the pathophysiology underlying OAB (Yamaguchi, 2002; Yamaguchi and Chapple, 2007; Peyronnet et al., 2019). Therefore, Trp64Arg polymorphism of *ADRB3*, leading to a missense mutation and altering receptor function, would be speculated on increased susceptibility to OAB (Ferreira et al., 2011; Meekins et al., 2019).

The result of two studies indicated that the Trp64Arg polymorphism was associated with susceptibility to OAB in Brazilian and Japanese populations (Ferreira et al., 2011; Honda et al., 2014). Ferreira et al. (2011) conducted a case-control study with 218 women in a Brazilian population. Genotyping of Trp64Arg polymorphism revealed that 51% of patients with OAB and 24.3% of controls with no OAB had the studied polymorphism. Similarly, Honda et al. performed a study with 201 women in a Japanese population Honda et al. (2014). The results revealed that 47% of the cases were either heterozygous or homozygous for Trp64Arg polymorphism, as compared with only 23% of controls. Meanwhile, another meta-analysis, including two studies, provided moderate credibility for associations of the Trp64Arg polymorphism with OAB (pooled OR, 2.5; 95%CI, 1.7–3.6; n = 419) (Qu et al., 2015). However, another study indicated that the Trp64Arg polymorphism was not susceptible to OAB in a Turkish population (Gurocak et al., 2015; Fırat et al., 2020). Gurocak et al. conducted a case-control study with 76 toilet-trained children older than 5 years of age from Turkey. Genotyping revealed that 12.5% of patients with OAB and 11.9% of controls had heterozygote mutation, and neither group had homozygote mutation Gurocak et al. (2015). Consistently, Fırat et al. (2020) performed a study with 120 women (1:1 matched), and the results indicated that 20% of the cases were either heterozygous or homozygous mutation, as compared with 18.4% of controls. However, the genotype distribution for *ADRB3* T > C polymorphism (ID: rs4994) was, in fact, departed from HWE with statistical significance in 60 healthy controls (*p* = 0.0003) (Moher et al., 2009). Therefore, the findings in this study will remain doubtful until they are replicated by investigation of other cohorts in accordance with HWE. Moreover, the results from a genome-wide association study (GWAS) discovered no significant association between OAB and any SNP (Funada et al., 2018). Although none of the SNPs validation or meta-analyses overlapped, this is common in GWAS, especially ones such as these, which have lower power (Funada et al., 2018). As a result, replication of association may require very large samples.

In our study, we carried out a meta-analysis to explore the overall effects of Trp64Arg polymorphism on OAB risk. The overall results of this meta-analysis demonstrated that there might be an association between Trp64Arg polymorphism and susceptibility to OAB in allele model, dominant model, and heterozygote comparison with a relative risk of 2.00 (95% CI 1.36–2.93), 2.13 (95% CI 1.20–3.76), and 2.07 (95% CI 1.13–3.79), respectively. The results indicated that the Trp64Arg polymorphism might be associated with OAB. However, in recessive model and homozygote comparison, no significant association between *ADRB3* Trp64Arg polymorphism and susceptibility to OAB was observed, with a relative risk of 2.47 (95% CI 0.63–9.73) and 3.12 (95% CI: 0.79–12.35), respectively. Future studies are needed to understand the associations between *ADRB3* Trp64Arg polymorphisms and susceptibility to OAB in these two conditions.

Random errors may cause misleading in our study. The samples in a meta-analysis should be at least as large as an adequately powered single trial. TSA may reduce the risk of random errors due to repetitive testing of accumulating data by evaluating meta-analyses not reaching the information size with monitoring boundaries. However, considering only four original studies and repetitive testing of accumulating data, meta-analyses are at risk of producing random errors and should not be trusted without TSA. We adjusted the meta-analysis with TSA that widened the CI (Brok et al., 2008; Wetterslev et al., 2017). Based on TSA, the results indicated that the association between Trp64Arg polymorphism and susceptibility to OAB turned out to be true positive in the allele model, even though there were merely four original studies. However, in the dominant model and heterozygote comparison, the results turned out to be false positive based on TSA. Meanwhile, in the recessive model and homozygote comparison, the results turned out to be negative based on TSA. Overall, the results of TSA indicated that we need more studies to identify the associations between *ADRB3* Trp64Arg polymorphisms and susceptibility to OAB, except for the allele model.

In this meta-analysis, there are some limitations which may influence the findings. First, based on previous studies, Trp64Arg polymorphism occurs with an approximate frequency of 12–20% in the Turkish population, 14–16% in the Caucasian population, and 50% in the Japanese and Brazilian populations with OAB. It is indicated that the effects of beta3-AR polymorphism vary between ethnicities (Abdellaoui et al., 2019). However, the subgroup analyses were not conducted according to ethnicity to explore the possible explanations for heterogeneity because of only four original studies. Second, due to the lack of original study reported frequencies and mode of inheritance of Trp64Arg in cases based on the severity of OAB, the genotype and allele frequency analyses were not performed for symptom severity (Gotoh et al., 2015).

Despite the limitations, the results of our meta-analysis suggest that Trp64Arg polymorphisms in *ADRB3* might increase the risk of OAB twice in the allele model. Meanwhile, further well-designed studies with large sample sizes are required to confirm the present findings in other conditions.

## Conclusion

Our analysis indicated that Trp64Arg polymorphisms in *ADRB3* might increase the risk of OAB twice in the allele model; however, further well-designed studies with large sample sizes are required to confirm the present findings in other modes and comparisons.

## Data Availability

The original contributions presented in the study are included in the article/Supplementary Material; further inquiries can be directed to the corresponding author.

## Supplementary Material

Supplementary Figure S1 Forest plots for the association between *ADRB3* gene polymorphism and susceptibility to OAB in the dominant model (CC + TC vs. TT).

Supplementary Figure S2 Forest plots for the association between *ADRB3* gene polymorphism and susceptibility to OAB in heterozygote comparison (TC vs. TT).

Supplementary Figure S3 Forest plots for the association between *ADRB3* gene polymorphism and susceptibility to OAB in the recessive model (CC vs. TT + TC).

Supplementary Figure S4 Forest plots for the association between *ADRB3* gene polymorphism and susceptibility to OAB in homozygote comparison (CC vs. TT).

Supplementary Figure S5 Result of TSA for the association between *ADRB3* gene polymorphism and susceptibility to OAB in the dominant model (CC + TC vs. TT).

Supplementary Figure S6 Result of TSA for the association between *ADRB3* gene polymorphism and susceptibility to OAB in heterozygote comparison (TC vs. TT).

Supplementary Figure S7 Result of TSA for the association between *ADRB3* gene polymorphism and susceptibility to OAB in the recessive model (CC vs. TT + TC).

Supplementary Figure S8 Result of TSA for the association between *ADRB3* gene polymorphism and susceptibility to OAB in homozygote comparison (CC vs. TT).
