# Supplementary figures and images for: Association Between Trp64Arg Polymorphism of Beta-3 Adrenergic Receptor Gene and Susceptibility to Overactive Bladder: A Meta-Analysis
Source: Front Genet. 2022 Jul 12;13:930084. doi: 10.3389/fgene.2022.930084 (PMC9315387; doi:10.3389/fgene.2022.930084)

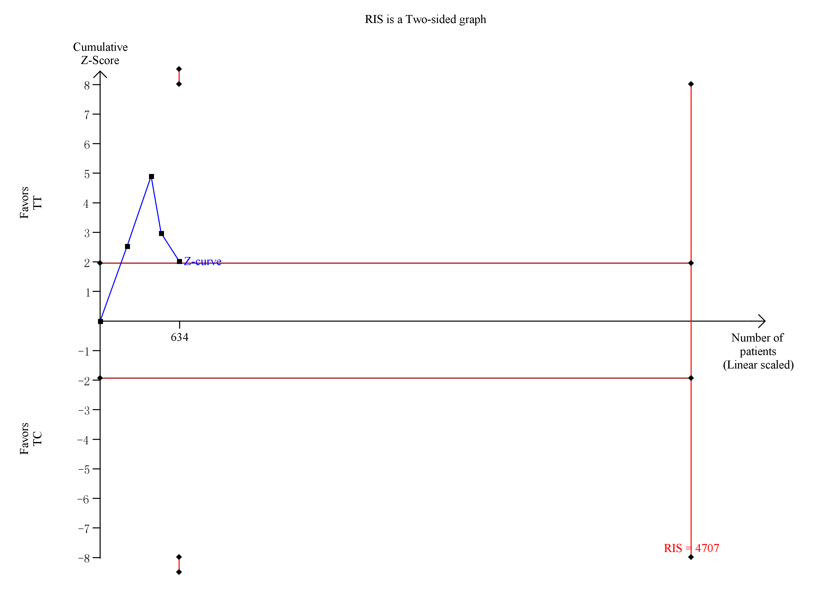

Supplement: Supplementary file 1 [file Image6.tif]

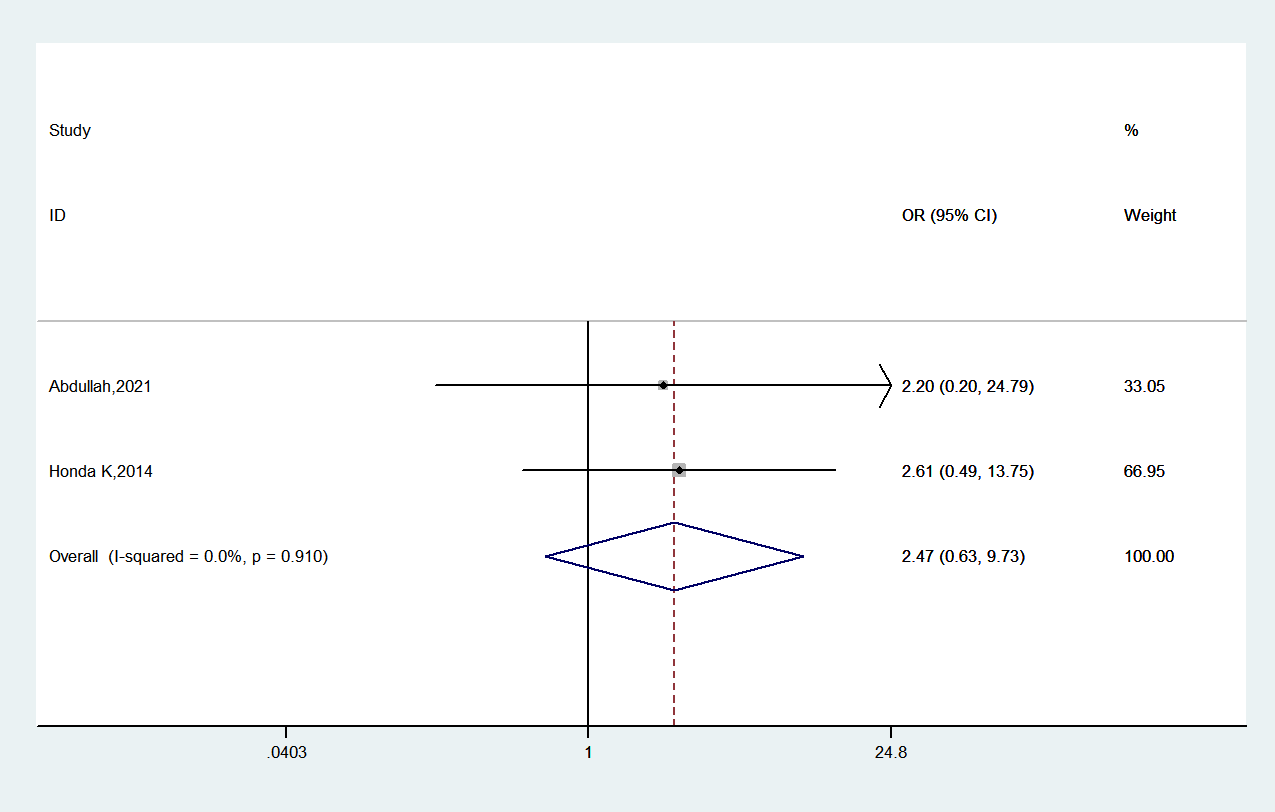

Supplement: Supplementary file 2 [file Image3.tif]

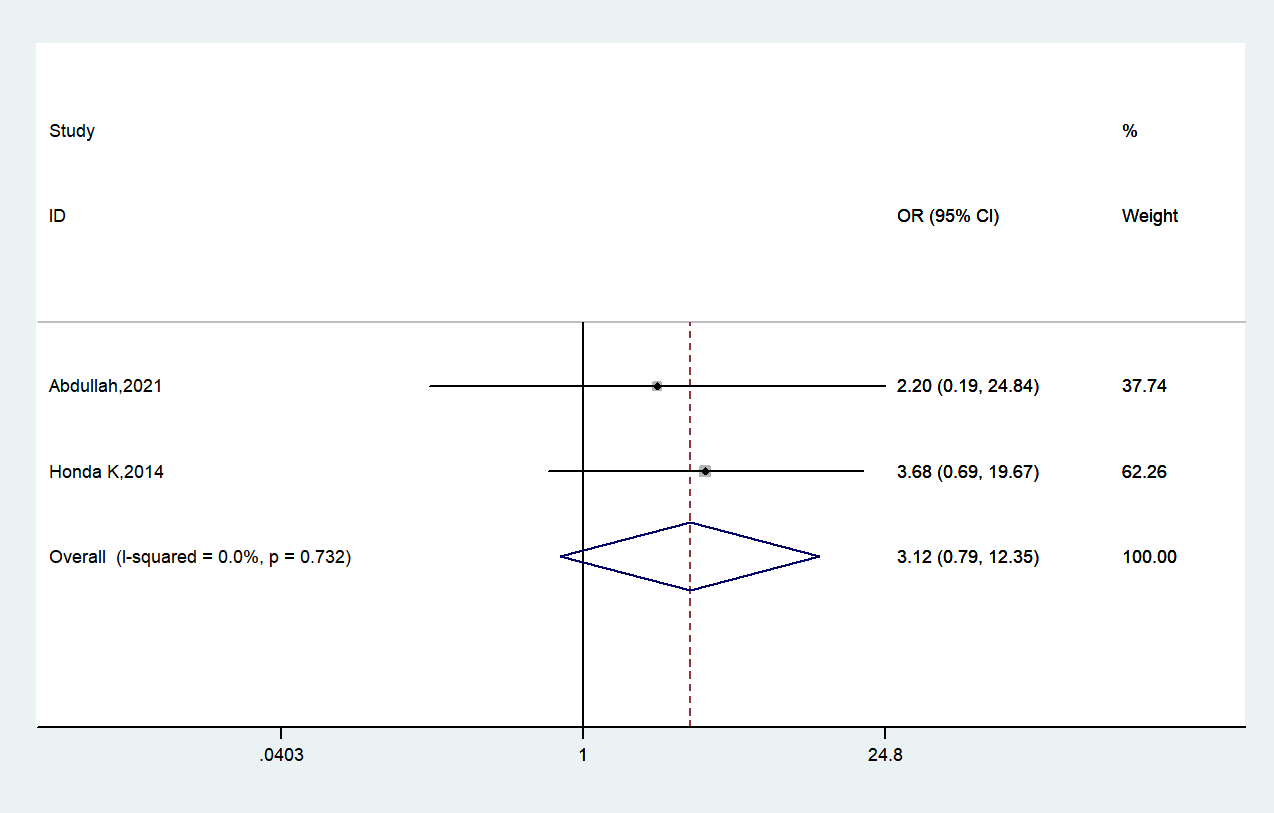

Supplement: Supplementary file 3 [file Image4.tif]

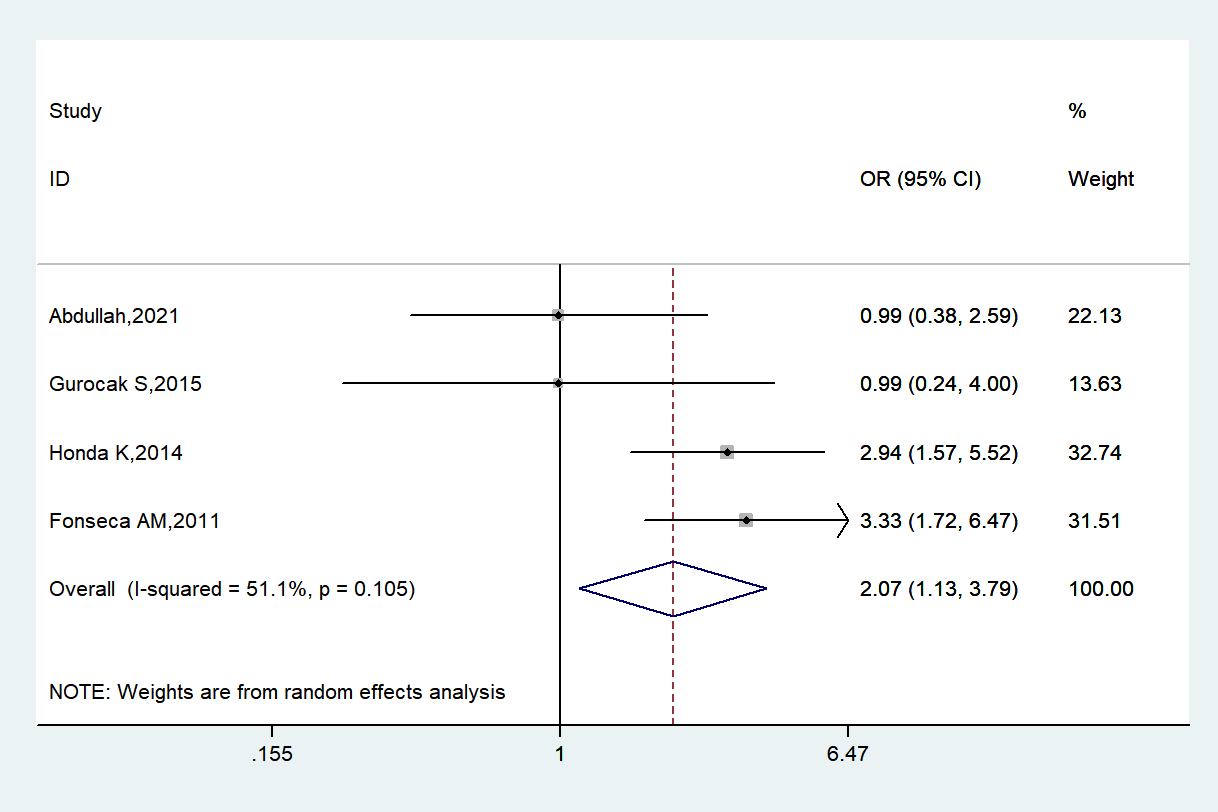

Supplement: Supplementary file 4 [file Image2.tif]

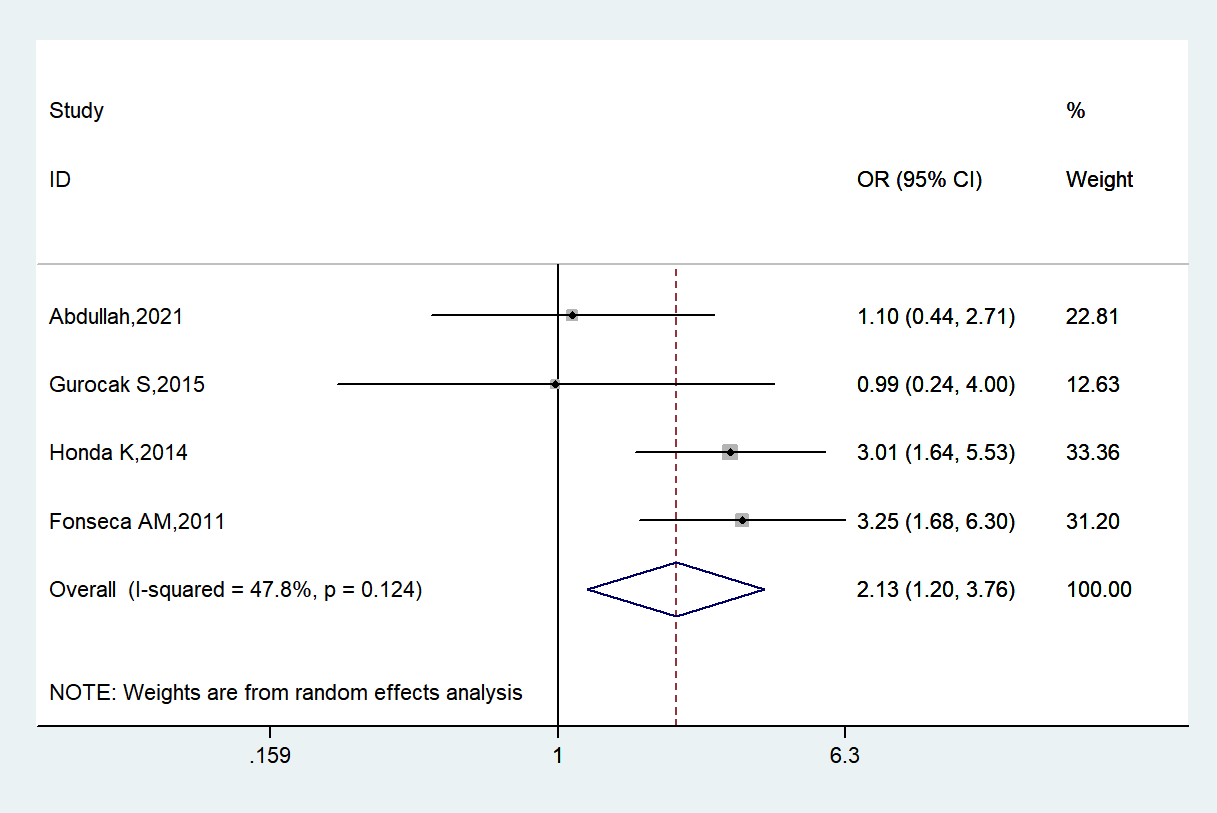

Supplement: Supplementary file 5 [file Image1.tif]

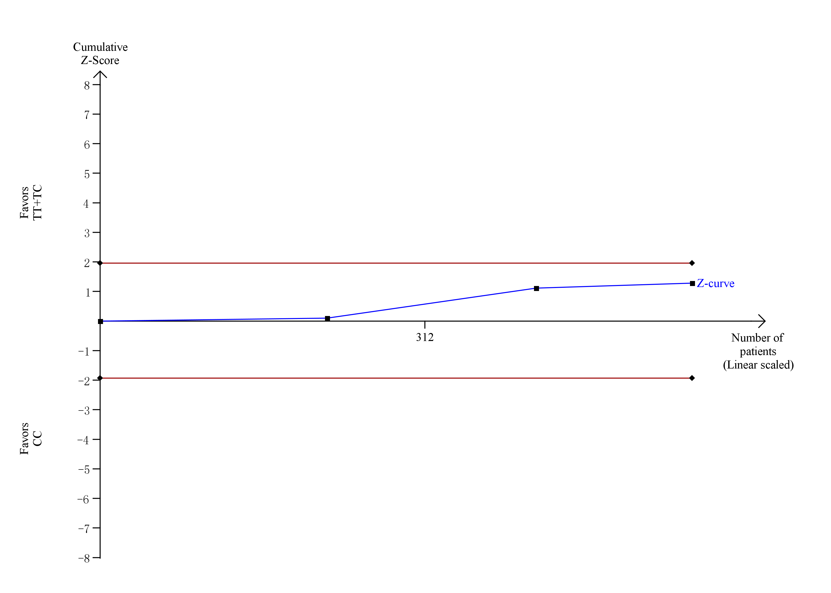

Supplement: Supplementary file 6 [file Image7.tif]

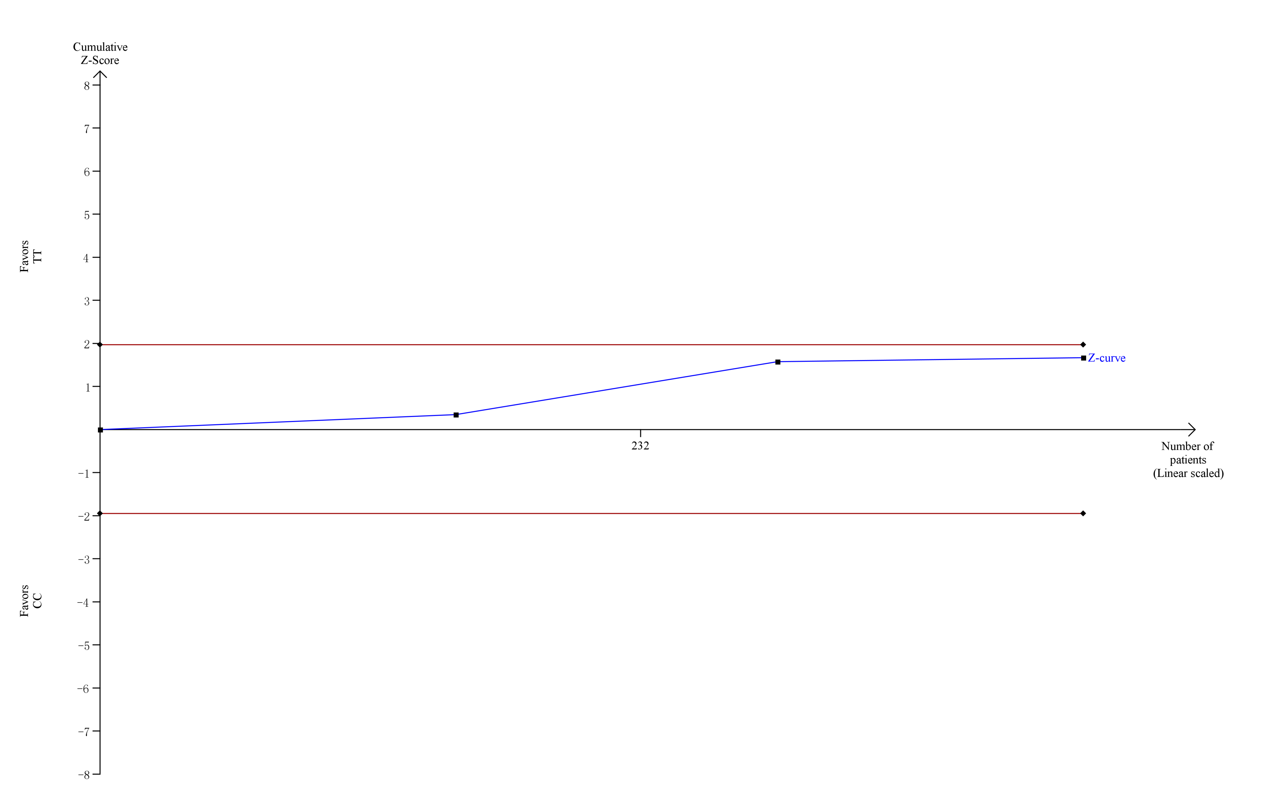

Supplement: Supplementary file 8 [file Image8.tif]

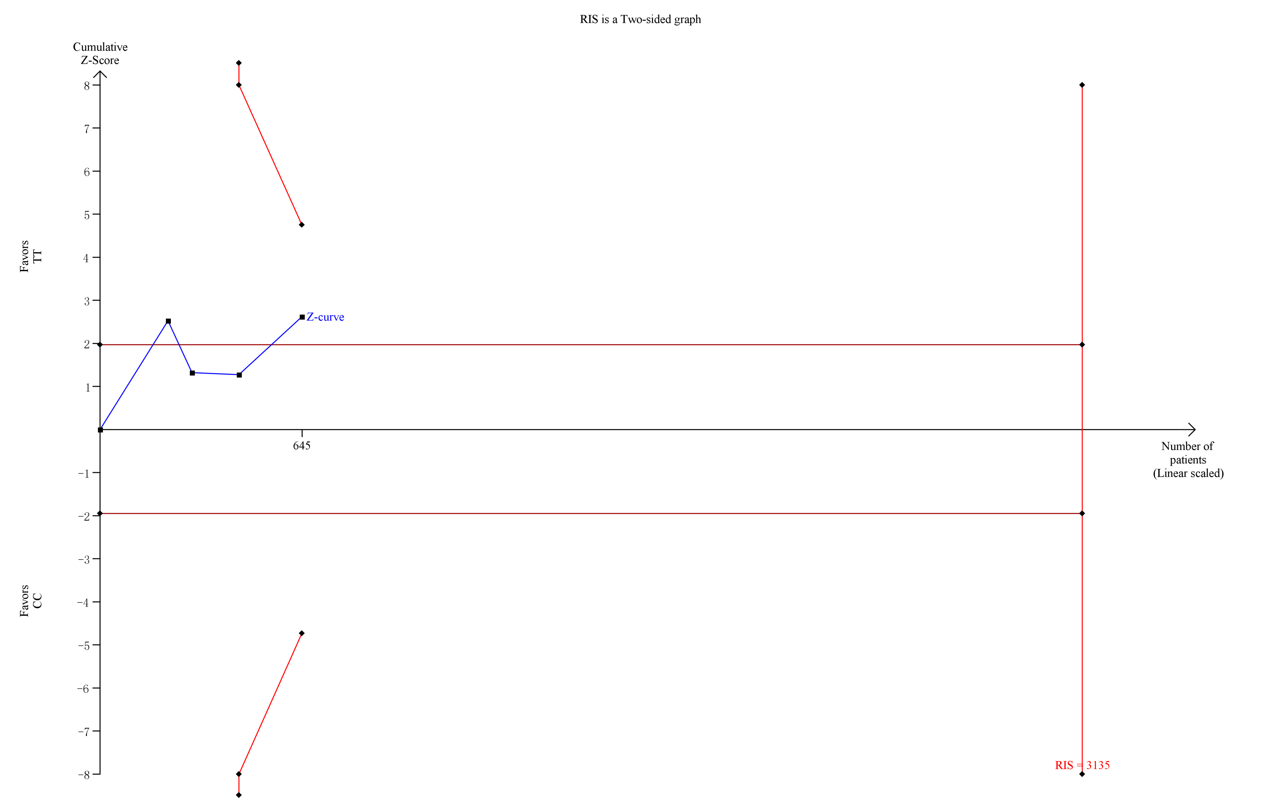

Supplement: Supplementary file 9 [file Image5.tif]
