# Supplementary material for: Association Between Trp64Arg Polymorphism of Beta-3 Adrenergic Receptor Gene and Susceptibility to Overactive Bladder: A Meta-Analysis
Source: Front Genet. 2022 Jul 12;13:930084. doi: 10.3389/fgene.2022.930084 (PMC9315387; doi:10.3389/fgene.2022.930084)
Supplement: Supplementary file 10 [file Table2.doc]

| **Table S2** Distribution of *ADRB3* SNP64 T>C genotype among OAB cases and controls | | | | | | | | | | | | | | | | | | | | |
| --- | --- | --- | --- | --- | --- | --- | --- | --- | --- | --- | --- | --- | --- | --- | --- | --- | --- | --- | --- | --- |
| corresponding author | Genotype | | | | | | | | | | | | | | | | |  | *P-*vaule of HWE | |
|  | Case | | | | | | | |  | Control | | | | | | | |  |  |  |
|  | TT | |  | TC | |  | CC | |  | TT | |  | TC | |  | CC | |  |  |  |
|  | n | % |  | n | % |  | n | % |  | n | % |  | n | % |  | n | % |  | Case | Control |
| Abdullah , 2021 | 61 | 84.7 |  | 9 | 12.5 |  | 2 | 2.8 |  | 67 | 85.9 |  | 10 | 12.8 |  | 1 | 1.3 |  | 0.04 | 0.39 |
| Gurocak S, 2015 | 30 | 88.2 |  | 4 | 11.8 |  | 0 | 0 |  | 37 | 88.1 |  | 5 | 11.9 |  | 0 | 0.0 |  | 0.72 | 0.68 |
| Honda K, 2014 | 53 | 53.0 |  | 42 | 42.0 |  | 5 | 5 |  | 78 | 77.2 |  | 21 | 20.8 |  | 2 | 2.0 |  | 0.36 | 0.68 |
| Fonseca AM, 2011 | 24 | 49.0 |  | 25 | 51.0 |  | 0 | 0 |  | 128 | 75.7 |  | 40 | 23.7 |  | 1 | 0.6 |  | 0.02 | 0.26 |
